# Therapeutic Effect of a Soft Robotic Glove for Activities of Daily Living In People With Impaired Hand Strength: Protocol for a Multicenter Clinical Trial (iHand)

**DOI:** 10.2196/34200

**Published:** 2022-04-05

**Authors:** Anke Ida Roza Kottink, Corien DM Nikamp, Foskea P Bos, Corry K van der Sluis, Marieke van den Broek, Bram Onneweer, Janneke M Stolwijk-Swüste, Sander M Brink, Nicoline BM Voet, Jacob B Buurke, Johannes S Rietman, Gerdienke B Prange-Lasonder

**Affiliations:** 1 Roessingh Research and Development Enschede Netherlands; 2 Department of Biomechanical Engineering University of Twente Enschede Netherlands; 3 Department of Biomedical Signals and Systems University of Twente Enschede Netherlands; 4 Reade Amsterdam Netherlands; 5 University of Groningen University Medical Center Groningen Department of Rehabilitation Medicine Groningen Netherlands; 6 Sint Maartenskliniek Ubbergen Netherlands; 7 Rijndam Rehabilitation Rotterdam Netherlands; 8 Department of Rehabilitation Medicine Erasmus MC Rotterdam Netherlands; 9 De Hoogstraat Rehabilitation Utrecht Netherlands; 10 Centre of Excellence for Rehabilitation Medicine Brain Centre Rudolf Magnus University Medical Centre Utrecht Utrecht Netherlands; 11 Department of Rehabilitation Medicine Isala Zwolle Netherlands; 12 Rehabilitation center Klimmendaal Arnhem Netherlands; 13 Department of Rehabilitation Radboud University Medical Center Donders Institute for Brain, Cognition and Behaviour Nijmegen Netherlands; 14 Roessingh Center for Rehabilitation Enschede Netherlands

**Keywords:** upper extremity, hand function, hand strength, robotics, rehabilitation, assistive technology, activities of daily living, wearable devices, soft-robotic glove, wearable, hand, robot, assist, protocol, therapy, support, intervention, function

## Abstract

**Background:**

Decline of hand function, especially reduced hand strength, is a common problem that can be caused by many disorders and results in difficulties performing activities of daily living. A wearable soft robotic glove may be a solution, enabling use of the affected arm and hand repeatedly during functional daily activities and providing intensive and task-specific training simultaneously with assistance of hand function.

**Objective:**

We aim to investigate the therapeutic effect of an assistive soft robotic glove (Carbonhand).

**Methods:**

This multicenter uncontrolled intervention study consists of 3 preassessments (T0, T1, and T2), a postassessment (T3), and a follow-up assessment (T4). Participants are patients who experience hand function limitations. For the intervention, participants will use the glove during activities of daily living at home for 6 weeks, with a recommended use of at least 180 minutes per week. The primary outcome measure is handgrip strength, and secondary outcome measures are related to functional arm and hand abilities, amount of glove use, and quality of life.

**Results:**

The first participant was included on June 25, 2019. Currently, the study has been extended due to the COVID-19 pandemic; data collection and analysis are expected to be completed in 2022.

**Conclusions:**

The Carbonhand system is a wearable assistive device, allowing performance of functional activities to be enhanced directly during functional daily activities. At the same time, active movement of the user is encouraged as much as possible, which has potential to provide highly intensive and task-specific training. As such, it is one of the first assistive devices to incorporate assist-as-needed principles. This is the first powered clinical trial that investigates the unique application of an assistive grip-supporting soft robotic glove outside of clinical settings with the aim to have a therapeutic effect.

**Trial Registration:**

Netherlands Trial Register NTR NL7561; https://www.trialregister.nl/trial/7561

**International Registered Report Identifier (IRRID):**

DERR1-10.2196/34200

## Introduction

Limitations in hand function are a common issue, with a diverse range of underlying causes. During an open population survey in Rotterdam, the Netherlands, 17% of respondents reported suffering from hand pain, and over 13% presented with hand disability [1]. One particularly debilitating aspect of hand function limitation is decreased hand strength, which occurs with a wide range of conditions, such as arthritis, trauma-induced hand injuries, neuromuscular diseases, orthopedic problems, and neurological disorders. For example, arthritis—inflammation of joints—comes in many forms, of which osteoarthritis, with 13% to 26% incidence [2], and rheumatoid arthritis, approximately 1% incidence [3,4], are most prevalent. Traumatic hand injury is even more common, with incidence rates reported between 57 to approximately 700 per 100,000 [5].

People suffering from loss of hand function experience marked difficulties grasping, holding, and manipulating objects [6] that subsequently lead to difficulties performing activities of daily living independently [7-9]. These limitations can have a negative effect on participation in society and quality of life [10-12]. Depending on the progressive or regressive nature of the limitations of the hand, hand strength and hand function can be maintained or even improved to a certain level of functioning through intensive exercise programs during inpatient or outpatient rehabilitation or community-based physical therapy. For example, strengthening, stretching, and joint mobility exercises are recommended for treating hand osteoarthritis [13]. For rheumatoid arthritis with hand involvement, physical therapy that consists of functional exercises for the hand, ideally integrated in task-specific activities and in a daily regimen, is recommended [14]. Evidence from a randomized controlled trial [15] showed that a tailored hand exercise program for adults with rheumatoid arthritis who had pain and dysfunction of the hands doubled the treatment effect on hand function, activities of daily living, work, satisfaction, and confidence in symptom self-management, in comparison with that of a good-quality usual-care control intervention consisting of joint protection advice and splinting. Similar recommendations are valid for hand limitations due to neurological disorders [16]. Nevertheless, many people suffering from impairments in hand strength do not regain the previous levels of function, even if they are actively involved in exercise programs, or they relapse as soon as they stop exercising [17]. Ideally, people suffering from loss of hand strength should be encouraged to use their affected hands daily within their abilities [14]. For those left with limited functional independence, all that remains is reliance on assistance for activities of daily living.

Assistive devices can be used to support activities that are hindered by physical limitations. Many assistive devices for activities of daily living are available [18,19]—from simple assistive tools (eg, knife with an adapted handle) to large robotic systems that can act as a substitute for activities performed by people themselves, in the case of very severe limitations (eg, a wheelchair-mounted robotic manipulator) [20,21]. Although the use of assistive technologies allows more autonomy, most often, the devices are a substitute for the function of the person [22-24], instead of stimulating active use of affected limbs. Patients using their affected hands as actively as possible are more likely to maintain or improve hand function; therefore, people have a strong desire to keep using their affected limbs as much as possible, and are not keen to use technology that overrides what little function remains [25].

Recent technological developments in the field of robotics facilitate direct support of motor function for prolonged periods and in environments beyond clinical centers. Soft robotic gloves, constructed of textiles and soft materials (sensors and artificial tendons) that are comfortable to wear and compliant with human movement and which can be used to optimize hand- and finger-related functional abilities, have become increasingly available in the last decade [26]. In a recent review [26], Proulx and colleagues concluded that soft robotic gloves seemed to be a safe and promising technology to improve dexterity and functional performance in individuals with reduced hand function as a result of a neurological event. However, the level of evidence for the effectiveness of these devices needs to be substantially increased before their use in daily life or into neurorehabilitation programs is recommended. One promising approach is robotics with assist-as-needed support, in which the support adapts to the abilities of the user. One particular development is a wearable soft glove (Carbonhand, Bioservo Technologies AG), to enhance a person’s grip during activities of daily living. Since the wearable device is equipped with assist-as-needed control and supports meaningful daily activities for the user, the glove not only acts as an assistive device but also provides active, intensive, and task-specific training. This facilitates full integration of the glove in functional activities, allowing a high dose of practice that is highly task-specific, without taking additional time out of the person’s daily schedule. Hence, it is possible that unsupported arm and hand function may improve after prolonged use of the glove in daily life. In a first clinical trial [27], in which one of the intervention groups used a previous version of this system, we discovered that 4 weeks of use of the soft robotic glove at home had a positive effect on hand strength, functional performance, and dexterity (assessed without the glove) in older adults suffering from rheumatoid arthritis or osteoarthritis and in stroke patients; however, the study was not powered sufficiently for a conclusive outcome. Therefore, we aim to investigate whether 6 weeks of use of a state-of-the-art grip-supporting soft robotic glove (Carbonhand) as assistive device during activities of daily living at home results in a therapeutic effect in patients with hand function problems. In accordance with the findings of our pilot study [27], we expect that 6 weeks of use of a grip-supporting soft robotic glove will result in increased grip strength, improved hand function, and increased hand function abilities in a broad population.

## Methods

### Study Design

**Figure 1:**
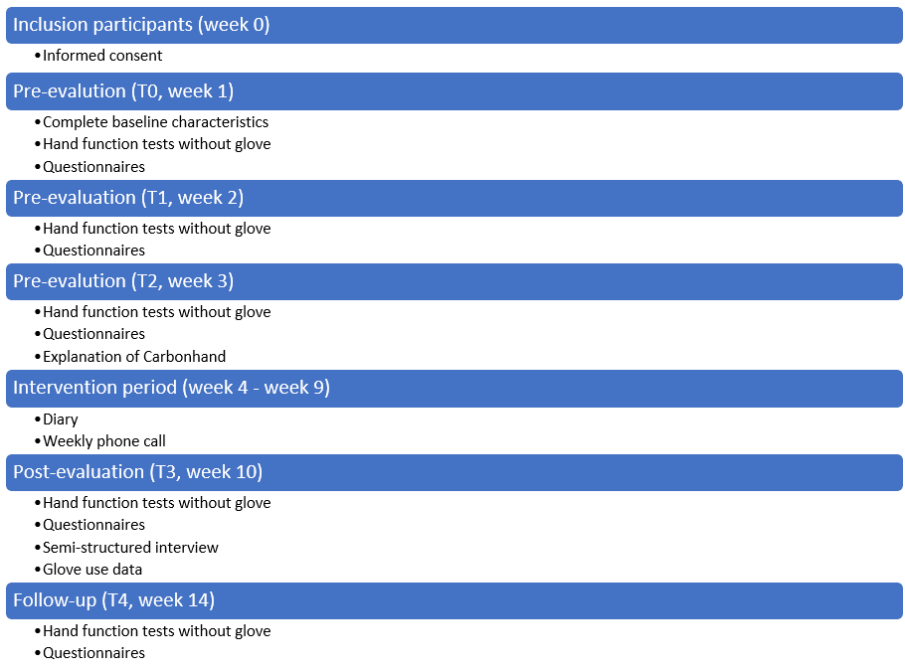
Study flowchart.

This study is a multicenter uncontrolled intervention trial (iHand). All participants will be assessed 5 times: 3 preassessments, 1 postassessment, and 1 follow-up assessment (Figure 1). SPIRIT (Standard Protocol Items: Recommendations for Interventional Trials) [28] will be used.

### Setting

The study takes place in 8 clinical centers (rehabilitation centers and rehabilitation departments of academic hospitals) in the Netherlands: Roessingh Centre for Rehabilitation in Enschede, University Medical Center Groningen, Isala in Zwolle, Rijndam Rehabilitation in Rotterdam, Reade in Amsterdam, De Hoogstraat Rehabilitation in Utrecht, Sint Maartenskliniek in Ubbergen, and Klimmendaal in Arnhem. Klimmendaal did not participate at the start of the project but was included as center in June 2021.

### Study Coordination

Roessingh Research and Development BV is the coordinator of the trial and responsible for the study design, management, data collection, and data analysis. Bioservo Technologies AB is the manufacturer of the Carbonhand system and the project manager and sponsor of the iHand project. Clinical Trial Service BV was contracted as external data monitor, to check that execution of the study in participating centers follows the study protocol and good clinical practice [29].

### Ethics

The protocol has been approved by the Medical Ethical Committee of Twente (NL68135.044.19); the Medical Ethical Committee of Twente recently merged with the Medical Research Ethics Committees United. The study was also registered in the Netherlands Trial Register (NTR NL7561). Since the study includes the use of a medical device outside of its intended use, the study is also registered with the Dutch Health and Youth Care Inspectorate. Although the risk of the study is classified as low, both patient and liability insurance have been obtained. All administrative and protocol-related amendments will be submitted for approval to the Medical Ethical Committee. After receiving their approval, these modifications will be reported to all participating centers and Clinical Trial Service BV via email and made available on a password-secured website (only the latest versions of all study-related documents are posted). If forms such as the study protocol or information letter are changed, the newest version will be sent to the participating centers and they will be asked to use this version from then on.

### Participants

We aim to enroll patients with chronic perceived hand function problems, including decreased handgrip strength. Since this impairment is caused by a wide range of disorders, such as acquired brain injury, osteoarthritis, rheumatoid arthritis, spinal cord injury, orthopedic problems or other neurological disorders, we have chosen not to limit the study to a single disorder but rather to focus on the common motor limitation, for which the intervention was developed. Therefore, the study sample is heterogeneous. In each center, a rehabilitation physician or clinical researcher is involved in the identification of potential participants for the study, based on screening of predefined selection criteria. The candidates will be contacted by the professional from that particular center to inform them about the study. When participants are interested, an information letter is sent to them. After 1 week, the health care professional will contact candidates to determine their interest in participating in the study and to answer possible questions. If interested in participating, a physical appointment is scheduled to obtain informed consent form, and then, to complete the screening procedure (some selection criteria require physical tests).

Inclusion criteria are (1) age between 18 and 90 years, (2) being in a chronic and stable phase of disease, (3) having received treatment for limitations in performing activities of daily living due to a decline in hand function (regardless of underlying disorder) at the involved rehabilitation center and department, (4) being capable of at least 10° of active extension of the wrist and fingers and 10° of active flexion of the fingers, (5) having the ability to make a pinch grip between thumb and middle or ring finger, (6) having the ability to put on the glove, (7) having sufficient cognitive status to understand 2-step instructions (judged by personal contact between participant and experienced health care professional), (8) living at home, and (9) providing written informed consent. To ensure that people are able to meet the recommended amount of use, an initial inclusion criterion was that the most affected hand of the participant was the dominant hand; however, due to support of the glove during mainly bimanual activities, this inclusion criterion was removed.

Exclusion criteria were (1) having severe sensory problems of the most affected hand, (2) having severe acute pain of the most affected hand, (3) having wounds on their hands resulting in a problem when using the glove, (4) having severe contractures limiting passive range of motion, (5) having comorbidities that limit functional use and performance of the arms and hands, (6) having severe spasticity of the hand (≥2 points on Ashworth Scale), (7) participating in another study that can affect functional performance of the arm and hand, (8) receiving arm or hand function therapy during the course of the study, or (9) having insufficient knowledge of the Dutch language to understand the purpose or methods of the study. Reasons for exclusion will be reported.

Eligible candidates participate in a test session, in which the glove system is explained, the size of the glove and straps are determined, and the support of the glove is tested by performing several grasps (10-15 minutes). The aim of this test session is to allow participants to experience the support that can be expected from the glove system and make a well-informed decision about the study.

### Carbonhand System

#### Overview

**Figure 2:**
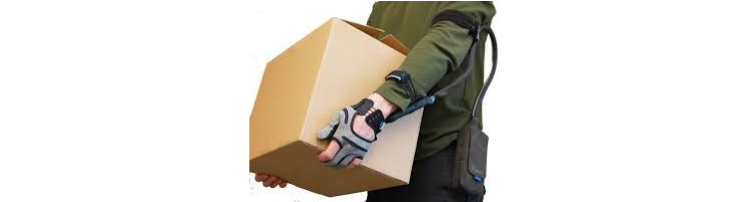
Carbonhand system.

The Carbonhand system is a soft robotic device, constructed of textiles and soft materials that are comfortable to wear and compliant with human movement. The glove enhances a user’s grip based on voluntary, active initiation (Multimedia Appendix 1). The glove is available in several sizes (extra small to extra large) and in both right-hand and left-hand versions. The total weight of the system is approximately 700 grams. Carbonhand is a CE-marked assistive medical device, but CE approval does not extend to the intended use in this study (therapeutic effect). The Carbonhand system consists of a glove and a control unit (Figure 2).

#### Glove

The main purpose of the glove is to apply the forces generated by the motors in the control unit and to provide the control unit with sensory input from touch sensors at the fingertips. The glove has a slim design and the same look and feel as a regular glove. Three fingers—the thumb, middle, and ring finger—are covered by the glove and are actuated by 3 separate motors to support power grip. The index finger and little finger are left uncovered; the index finger is left uncovered to allow tactile sensing. Actuation finger flexion is triggered by an interaction force between the fingertips and an object through pressure sensors sewn into the glove at the tips of the 3 actuated fingers. The forces are applied by artificial tendons that are sewn into the glove along the length of the fingers, which induce flexion of the fingers when contracted.

#### Control Unit

The control unit contains a battery, 3 motors, and a microcontroller. It is worn at the waist, on the hip or on the back of the user, using a clip or belt. A cable connects the control unit with the glove, via a detachable connection located close to the glove. An upper and lower arm strap, both available in different sizes, lead the cable along the arm. Embedded software in the control unit proportionally adjusts the amount of assistive force to help the user close the hand—an increase of force induced by the user and recorded at the fingertips will increase the force applied by the actuators and relaxation of active grip reduces the interaction force recorded by the fingertips, resulting in a gradual decline in force supporting finger flexion. Via a smartphone app, the sensitivity and the amount of force produced by the actuators can be adjusted for each finger by the health care professional, and the configuration—which sensors activate which fingers—can be set (eg, activation of the sensor at the middle finger can be set to actuate both the middle and the ring finger simultaneously). Specific useful combinations of a certain amount of sensitivity, force, and configuration of fingers can be saved as profiles. Profiles can be created for specific activities (eg, carrying heavy objects or grasping small objects such as a paintbrush) or general purposes (eg, a low, medium, or high amount of force, to allow the user to switch during the day). Profiles are individually created by the health care professional in consultation with the user, depending on the individual situation and needs. A maximum of 3 custom-designed profiles can be saved under a specific name at the control unit’s buttons for use by the patient at home.

The control unit can be used for a new participant after the previous participant has completed the intervention. For hygienic reasons, each participant will receive a new glove. Because the glove is not washable, due to the integrated electronics, participants are advised to wear a rubber household glove on top of the Carbonhand glove during activities that may expose the hand to liquids or dirt. The rubber household glove is also provided to the participant.

### Baseline Characteristics

Participant characteristics—age, gender (male, female, nonbinary), impairment or diagnosis, time since diagnosis, most affected side, and dominant side—will be collected from the medical record or from the patient by the health care professionals. 

### Study Procedure

All health care professionals involved in the study received extensive training prior to the start of the patient recruitment in order to standardize the execution of the study across the different centers. Plenary instruction sessions were scheduled; health care professionals were trained in good clinical practice, execution of the study protocol, in fitting and operating the Carbonhand system, and in explaining (following a standard procedure) the use of the Carbonhand system to the participants. All instructions were also documented in logbooks and manuals, which were provided to the professionals. The latest versions of these documents are maintained on a secure project website.

In total, there are 5 assessments for each participant. Three preassessments (T0, T1, and T2) are scheduled across 3 weeks, as baselines, directly prior to the intervention period. After completing the third baseline session, the Carbonhand system is manually adjusted by the professional to the individual participant. Attention is paid to the correct size of the glove and arm straps, the finger length of the glove, and creating 3 support profiles. In addition, instructions about all aspects of the Carbonhand system are given, demonstrated, and practiced with the participant, until the professionals are confident that the participant knows how to use the system properly at home. In addition to receiving the Carbonhand system, all participants will be provided with a short user manual (in Dutch) and an excerpt from the exercise book [30], in which exercises (functional activities that can be used in the home situation) are described for people with almost complete function of the arm.

During the 6-week intervention period, participants use the Carbonhand system at home. Within 1 week of the end of the intervention period, postassessment (T3) is conducted; 4 weeks later, a follow-up assessment (T4) is conducted, to measure the retention of effects.

Monitoring visits by an independent study monitor are planned for each participating center to ensure the continued protection of participants rights and well-being, to assure protocol adherence, and to verify data integrity during the study in compliance with good clinical practice. All protocol deviations will be filed. We anticipate having 5 monitor visits to each center during the course of the study.

### Intervention

Participants wear the Carbonhand glove on their most affected hand. If glove use during the 6-week intervention period is interrupted by unforeseen circumstances (eg, being ill for a few days), the participant is allowed to extend this period to achieve 6 weeks of glove use. The participants are free to choose for which activities, when, and for how long they use the Carbonhand system. However, we will recommend using the system at least 180 minutes per week for 6 weeks during the most common activities of daily living [31], such as lifting and carrying items, performing hobbies, cleaning cooking, and gardening. The recommended intensity of use is based on the findings of a systematic review [32]—a minimum dose of at least 16 hours (which is equal to 960 minutes) is needed to reach a functional effect in the stroke population. During the period of home use, the health care professional has weekly contact by phone to ask about the participants’ experience with the Carbonhand system, to verify adherence to the intervention, to ask if adjustments need to be made to the Carbonhand system’s support profiles, and to respond to any potential problems (eg, device deficiencies, adverse events and serious adverse events) that might arise. Extensive notes are made by the health care professional during these phone calls. When adjustments need to be made to the support profiles on the Carbonhand system during the intervention period, an extra visit (either the participant to the center or the investigator to the participant’s home) will be scheduled. For each participant, all adverse events and serious adverse device effects are reported by the site investigators in the investigators site file and the web-based clinical database during the entire study period. Roessingh Research and Development BV is responsible for informing the Medical Ethical Committee about the occurrence of serious adverse device events.

### Outcome Measures

#### Overview

The primary outcome measure of the study is maximal handgrip strength. Secondary outcome measures, related to arm and hand function, functional ability, amount of glove use, pain, and quality of life (Table 1), are only performed on the most affected side. All assessments are estimated to take 1.5 to 2 hours.

During the intervention period, the amount of glove use will be recorded automatically by the Carbonhand system. In addition, participants are asked to keep a diary about daily use of the glove and all activities in which they engage. In order to get a thorough understanding about the experience of the participants of using the Carbonhand system during activities of daily living at home and the user friendliness of the system, semistructured interviews will take place after the intervention period (T3).

**Table 1:** Overview of outcome measures.

Outcome measure	International Classification of Functioning, Disability and Health component	Domain	Assessment
Maximal handgrip strength	Body function	Grip strength	T0-T4
Maximal pinch strength	Body function	Pinch strength	T0-T4
Static grip endurance	Body function	Grip endurance	T0-T4
Action Research Arm Test	Activity	Upper extremity performance	T0-T4
Jebsen-Taylor hand function test	Activity	Fine and gross hand motor skills	T0-T4
Michigan Hand Outcomes Questionnaire–Dutch version	Activity	Self-perceived health state	T0-T4
Motor Activity Log	Activity	Self-perceived upper limb performance	T0-T4
Numeric pain rating scale	Body function	Self-perceived intensity of pain	T0-T4
EuroQol 5 dimension, 5 level	Participation	Self-perceived health-related quality of life	T0-T4
Short-form 36	Participation	Self-perceived quality of life	T0-T4
Glove use data	Activity	Registered amount of use	Intervention period
Diary	Activity	Self-perceived amount of use	Intervention period
Semistructured interview	Activity	Patient’s experiences of Carbonhand use	T3

#### Primary Outcome Measure

Assessment of maximal handgrip strength will be performed in accordance with American Society of Hand Therapist guidelines [33]: with the participant sitting comfortably and in an upright position with the elbow of the affected arm close to their body, flexed at a 90° angle, and holding the dynamometer (Jamar hydraulic hand dynamometer, Patterson Medical) in their hand. The other parts of the body are not allowed to move or help to give more strength. The handle position of the dynamometer is adjusted for each participant, so that the middle phalanx of the middle finger is at 90° to the handle. The examiner will provide the participant with standardized verbal instructions. Participants will perform 3 maximal contractions for 5 seconds, while the examiner gently supports the base of the dynamometer. A 60-second-duration rest is taken between each contraction. If the third value is higher than the first and second, a fourth attempt will be added. This will be continued until the last value is lower than the second-to-last value. The mean value of the last 3 attempts will be used as the test score [33].

#### Secondary Outcome Measures

Maximal pinch strength will be assessed with the Baseline Lite Hydraulic Pinch Gauge dynamometer (Fabrication Enterprises). The pinch strength will be measured in 3 configurations—between the index finger and the thumb, the middle finger and the thumb, and the ring finger and the thumb—with the participant sitting in a straight-backed chair without arm supports, the elbow flexed at 90° and close to the body, the forearm in a neutral position, and the wrist in a neutral position or with slight extension (0º-30º) [33]. The pinch gauge is grasped with the distal segment and ventral side of the thumb and finger, while the pinch meter is slightly supported by the examiner. The examiner will provide the participant with standardized verbal instructions. Three maximal isometric contractions will be performed for at least 5 seconds, with a 60-second-duration rest period between each contraction. If the third value is higher than the first and second, a fourth attempt will be added. This will be continued until the last value is lower than the second-to-last value. The mean value of the last 3 attempts will be used as the test score [33].

Static grip endurance is measured in the same way as handgrip strength; however, this test determines endurance during a static hold. Participants will be instructed to squeeze and hold the Jamar hydraulic hand dynamometer with full effort for 30 seconds. Participants will not be informed of the time remaining. Relative endurance for this test was calculated as (*mean force during last second*) / (*mean force during first second*), and larger numbers reflect greater relative endurance (ie, less fatigue) [34].

The Action Research Arm Test is a reliable, valid, and sensitive measurement for dexterity that evaluates 19 tasks for distal and proximal arm motor function and is divided into 4 subscales: grasp, grip, pinch, and gross arm motor function [35,36]. The quality of performance on each item is rated on a 4-point ordinal scale that ranges from 0 (can perform no part of test) to 3 (performs test normally). The maximum score of the Action Research Arm Test is 57 points and will be scored as described in [35].

Jebsen-Taylor Hand Function Test is a reliable and valid test to evaluate functional hand motor skills in different patient groups and healthy people of various ages [37]. The test consists of 7 different unilateral hand skill tasks related to activities of daily living: (1) writing a sentence of 24 characters, (2) turning over 7.6 cm × 12.7 cm cards, (3) picking up and moving small common objects (eg, paper clips, coins, and bottle caps), (4) stacking checkers (test of eye–hand coordination), (5) simulated feeding (eg, teaspoon with beans), (6) picking up large empty cans, and (7) moving weighted (450 g) cans. The duration of each task will be recorded in seconds and summed for the test score [38].

Michigan Hand Outcomes Questionnaire–Dutch Language Version (a total of 57 items) assesses patients’ opinions their hands and health [39,40]. The questions are used to assess out how patients feel and to what extent they are capable of carrying out daily activities in the past week (with the exception of part 3, in which the last 4 weeks is used). The problem is mapped out within 6 domains: total hand function, activities of daily living, work situation, work performance, pain, aesthetics, and satisfaction. Within each domain, items are scored on a 5-point Likert scale. The scores are normalized to a range of 0 to 100. For the pain scale, higher scores indicate more pain. For the other 5 scales, higher scores indicate better performance.

The Motor Activity Log [41] is a semistructured questionnaire that assesses self-perceived amount of use and quality of movement of the affected arm and hand by stroke patients during activities of daily living. This questionnaire consists of 26 activities and has excellent test-retest reliability for both scores of each activity; each activity is rated by the participant for quality of movement and amount of use of the upper extremity on a 5-point scale.

An 11-point numeric pain rating scale—from 0 (no pain) to 10 (the most intense pain imaginable)—is used to measure the subjective intensity of pain (patients select a value that is most in line with the intensity of pain that they have experienced in the last 24 hours). The numeric pain rating scale has good sensitivity, while producing data that can be statistically analyzed [42].

EuroQol 5D-5L (EQ-5D) index is a standardized instrument that is applicable to a wide range of health conditions and treatments [43]. The first part records self-reported problems in mobility, self-care, usual activities, pain and discomfort, and anxiety and depression domains. Each domain is divided into 5 levels of severity, corresponding to no problems, slight problems, moderate problems, severe problems, and extreme problems. The second part comprises a visual analog scale from 0 (worst imaginable health state) to 100 (best imaginable health state). The EQ-5D is designed for self-completion by respondents. In this study, the EQ-5D will be used to calculate a preference-based summary index, based on time trade-off techniques, for which the value 0 represents death and 1 represents perfect health.

Short Form 36 is a 36-item valid and reliable questionnaire to assess the participants’ health perception [44]. The questionnaire consists of multi-item dimensions about the physical and mental well-being of the participant. The total and component scores, physical component summary (average score of the domains physical functioning, physical role functioning, bodily pain and general health), and mental component summary (average score of the domains vitality, emotional role functioning, social functioning and mental health) will be calculated. The will be converted to a scale from 0 to 100, where a higher score indicates a better quality of life [45].

Glove-use data will be automatically recorded by the Carbonhand system, such as total time the system is turned on (hours), average session length (minutes), cumulative number of grasps (for thumb, middle, and ring fingers separately), average frequency of grasps per minute (for thumb, middle, and ring fingers separately) and the average grasp force provided by user (for thumb, middle, and ring fingers). The parameters are extracted from the control unit after the 6 weeks of home use using a USB-connection and dedicated Carbonhand software (version LB.09.07; Bioservo Utility Public) by each participating center.

A diary will be kept daily by the participants during the 6-week intervention period. Participants will be asked to report how often and for how long they use the Carbonhand system each day, when they use the system (morning, afternoon, evening), and during which activities. Information from the diaries will be transcribed by the investigator. The study coordinator will perform thematic analyses on those transcriptions to extract qualitative information about glove use and participants’ experiences using the Carbonhand system.

A semistructured qualitative interview with open-ended questions will be performed by the investigator at the T3 assessment, to collect participants’ experiences about using the Carbonhand system in daily life and the user-friendliness of the system. The interview will last approximately 15 minutes and was developed by Roessingh Research and Development BV in collaboration with Bioservo with the aim of learning about the user experiences and to improve the Carbonhand system. Data extraction (written answers of the interview) will follow the same procedure as that used for the diaries.

If a participant withdraws from the study during the intervention period, we will aim to obtain the reason for stopping, the maximum handgrip strength, and glove-use data and to complete the semistructured interview.

### Sample Size

Based on a previous study [27], a mean improvement of 2.16 kg, with an estimated standard deviation of 5.2 kg, in handgrip strength is expected; therefore, a minimum of 56 participants (using power=0.8 and α=.05) are needed. When accounting for 10% dropout, a minimum of 63 participants are needed. Given that 7 participating centers are involved, each center aims to include 9 participants, but centers are allowed to include more if they can, up and until the total target sample size is achieved.

### Data Management

Prior to the start of the study, a data management plan that covers all aspects of handling data gathered during and after completion of the iHand project, such as collection, storage, back-up, documentation, access, sharing, reuse, preservation, and archiving (both at the study coordinator’s site and participating centers), was devised.

All documents related to this study and all participant data will be collected in the investigators’ site files. These files, containing personal and contact information, as well as the screening information from potential candidates, will be safeguarded at the participating center of the corresponding participants. All relevant data (participants’ characteristics and clinical assessments) will be copied from the case report form into a web-based clinical database for case report form data (Castor, Castor EDC) by site investigators using unique and anonymous participant codes. All questionnaires (Michigan Hand Outcomes Questionnaire–Dutch Language Version, Motor Activity Log, numeric pain rating scale, EQ-5D, and Short Form 36) are filled in by participants via the web-based clinical database directly on site (ie, these data are not stored in site files). One center will deviate from this procedure—the participants will fill in the questionnaires on paper. These forms are kept in the site file and the data will be entered into the Castor database by the investigator. Transcripts of the weekly phone calls with the participants during the intervention period, copies of the diaries kept by participants, and extracted glove-use data (.csv format) are sent to the study coordinator by email, with participant identification code, after completion of the study. These data will be stored in a secure location on the local computer network of the study coordinator. Personal data of the participants recruited by a particular center will not be reported in the database or in any communication beyond the center and are only accessible by the investigators from that center. If necessary, the participant can be linked to these data by a participant identification code list, which is safeguarded at each center separately. In the source document agreement, the location of personal data is described by each individual center. Participating centers only have access to their own data set. The study coordinator and monitor have access to the full data set.

All coded data will be stored for 15 years within the clinical database environment and a copy of that database will be stored at Roessingh Research and Development BV for an unlimited time period, backed up daily, after downloading the completed and closed data set from the database.

### Statistical Analysis

Outcome measures will be analyzed using SPSS statistical software (version 19; IBM Corp). Data from the 3 baseline assessments (T0, T1, and T2) will be averaged (for overall preassessment values). Statistical analysis of the therapeutic effect will be performed on all participants who completed all assessments up to and including T3 and T4. Data from participants who drop out before or during the intervention period will be analyzed separately to analyze the patient characteristics of dropouts, including the reasons for dropout, to identify potentially relevant information regarding reevaluation of target population, device-related issues, to inform proper interpretation of study outcomes.

Normality of data distribution for each outcome measure will be checked by visual inspection and with the Shapiro-Wilks test, prior to analyzing outcome measures. Descriptive statistics will be used for all outcome measures (mean and standard deviation or median and interquartile range as applicable). The overall level of significance will be set to *P*<.05.

In order to assess the effect of the intervention over time, linear mixed models will be used to analyze changes in outcome measures over time. If a significant difference is found between sessions, multiple comparisons are performed with Sidak posthoc analysis. If data are not normally distributed, logarithmic transformation will first be applied to potentially achieve normally distributed data and enable use of linear mixed models for analyses. Otherwise, the Friedman test will be used for nonparametric analysis.

In addition, correlations (Pearson correlation coefficient or the nonparametric Spearman correlation coefficient, depending on data characteristics) between amount of use (measured by diary, Carbonhand system, or Motor Activity Log) or baseline patient characteristics and change in hand function outcome measures will be calculated to evaluate whether an increased dose or specific patient characteristics are associated with better outcomes

## Results

The study started in June 2019. The first participant was enrolled on June 25, 2019. As of October 2021, we have enrolled 52 participants. As of March 2022, the study is still ongoing due to the COVID-19 pandemic and related restrictions. We expect data collection to be completed in 2022.

## Discussion

We aim to conduct a high-quality powered clinical trial, to investigate changes in unsupported hand function after 6 weeks of use of a grip-supporting soft robotic glove during activities of daily living by patients with hand weakness and hand function limitations. It is known that several weeks of training using a robotic device improves activities of daily living performance, hand function, and hand strength to a similar extent as conventional training in stroke and other neurological conditions [46]. Yet, robot-assisted training has not been investigated substantially in disorders such as osteoarthritis, rheumatoid arthritis, neuromuscular diseases, even though the training principles share common ground—providing high-dose exercise training to improve hand function. It is conceivable that robot-assisted training could have a positive effect in a wider range of disorders. Nevertheless, exercise therapy might not be enough to halt deterioration over time or to regain hand function to a level required to achieve independence in activities of daily living.

To support activities of daily living that remain very challenging or impossible, assistive technologies can be used. Assist-as-needed principles have been increasingly incorporated in both assistive and therapeutic technology [47]. The Carbonhand system is one such example that allows the performance of functional activities to be enhanced directly while using the affected arm and hand repeatedly during daily activities, to provide high-dose task-specific training. This combination allows a unique approach, extending direct support of activities of daily living with the possibility to stimulate improvement of hand function over time outside of a clinical setting. Findings may allow high doses of training throughout the day into people’s homes, in the most functional task-specific way possible, and possibly prevention of learned nonuse.

To the best of our knowledge, comparison with other studies will be difficult, because this is one of the first user trials that will apply and test a fully wearable robotic system to support hand function at home for unsupervised use during an extended period of multiple weeks. Moreover, other studies [26] that do examine effects of a soft robotic glove focused on examining the direct assistive effect of the glove by comparing performance with and without the glove.

One strength of this powered study is that the sample size calculation was based on results from a previous clinical study [27] that evaluated a similar assistive device. In addition, experiences from the previous study [27]were used in defining the broad range of data collected covering all levels of the International Classification of Functioning, Disability and Health [48], including grip and pinch strength measurements, functional ability measurements of the arm and hand, pain and quality of life questionnaires, semistructured interviews, diaries, and glove-use data. Another strength is the focus on the generalizability of the outcomes for patients with reduced hand strength, by including patients with a wide range of disorders from multiple centers. As a result, we expect that the results of this study will contribute to clinical practice, by identifying the role that assistive devices can play within the rehabilitation process of a wide range of patient groups. In addition, we pay particular attention to protocol adherence and data collection: all professionals involved in the study received extensive training to standardize the execution of the study in the different centers, in the use of the web-based clinical database for data collection, and in fitting and operating the Carbonhand system. Additional plenary instruction sessions for good clinical practice were scheduled. Each of those aspects are explained in detail in instruction manuals, which are available to all involved persons through a website. In addition, visits to each participating center are conducted by an independent monitor to ensure protocol adherence and data integrity. In this paper, we use the SPIRIT-checklist for transparency and completeness in reporting all key elements. Furthermore, dissemination of the study results is planned through presentations at both scientific and clinical conferences and publications in peer-reviewed scientific medical journals, to reach both health care professionals (medical doctors, occupational and physical therapists) and the academic community. The Vancouver Convention is used as guideline to determine authorship and no professional writers will be involved [49]. Upon reasonable request, data will be available from the corresponding author. In addition, press releases will be issued to web-based media and health care magazines with lay summaries of study outcomes to inform potential end users.

In addition to these strengths, we face some challenges. First, the therapeutic effects of Carbonhand will be assessed using an uncontrolled design because of practical reasons—limited availability of resources in terms of project funding and project duration. Second, some outcome measures, for example those assessed with the Motor Activity Log, Michigan Hand Outcomes Questionnaire–Dutch Language Version, Short Form 36, EQ-5D, and the semistructured interview and diaries, are self-reported, which includes the risk of socially desirable answering. Potential bias is reduced as much as possible by allowing participants to complete the questionnaires by themselves, without interference from the health care professional. Finally, participants that might be disappointed about the effect of the glove may have a higher risk of dropout during or directly after the intervention period. This may influence postintervention (T3) and follow-up (T4) results. In order to prevent this effect as much as possible, professionals were instructed to explain the importance of completing the study to all participants at the start of the study. In addition, the characteristics of the dropout sample will be analyzed to inform proper interpretation of study outcomes. Another limitation is the cumulative collection of glove-use data via the Carbonhand system. This means that the exact amount of use per day per week or per bout of activity cannot be retrieved. In order to obtain insight into day-to-day use of the glove, participants are asked to note this information daily in a diary.

This is the first powered clinical trial to investigate the unique application of an assistive grip-supporting soft robotic glove for use outside of clinical settings with the aim to have a therapeutic effect. Despite the abovementioned challenges, the study will provide a solid knowledge base about the therapeutic effect of 6 weeks of home use of an assistive grip-supporting glove.

## Appendix

Multimedia Appendix 1 General impression of the application of the Carbonhand system.

Multimedia Appendix 2 Peer Review Reports.

## Abbreviations

EQ-5D: EuroQol 5 dimension, 5 level
SPIRIT: Standard Protocol Items: Recommendations for Interventional Trials
