# Supplementary material for: Therapeutic Effect of a Soft Robotic Glove for Activities of Daily Living In People With Impaired Hand Strength: Protocol for a Multicenter Clinical Trial (iHand)
Source: JMIR Res Protoc. 2022 Apr 5;11(4):e34200. doi: 10.2196/34200 (PMC9019626; doi:10.2196/34200)
Supplement: Multimedia Appendix 2 [file resprot_v11i4e34200_app2.pdf]

Associated with document Ref. Ares(2017)2494441 - 16/05/2017

Proposal Evaluation Form

EUROPEAN COMMISSION

Horizon 2020 - Research and Innovation Framework Programme

Evaluation Summary Report

**Call:**	H2020-SMEINST-2-2016-2017
**Funding scheme:**	SME-2
**Proposal number:**	778274
**Proposal acronym:**	i-Hand
**Duration (months):**	24
**Proposal title:**	The First Soft Robotic Glove for Hand Injury Prevention and Rehabilitation
**Activity:**	SMEInst-01-2016-2017

N.	Proposer name	Country	Total Cost	%	Grant Requested	%
1	BIOSERVO TECHNOLOGIES AB	SE	3,169,260	100.00%	2,218,482	100.00%
	Total:		3,169,260		2,218,482	

Abstract:
i-Hand is a soft robotic glove that can add extra strength to the grip and increase grip function for a) persons who perform intense manual labor/sports and want to prevent any hand issues, b) persons who want to reduce the risk of occupational injuries related to manual activities at work or home and c) persons recovering from hand problems. The first generation of the glove (SEM glove) generated €1M of sales an average price of 4100 Euros in 2016. The second generation of the glove (i-Hand) is a modular platform that can be used to develop different product variants tailored for different markets, and that has been optimized for volume production. This is a disruptive innovation for medical rehabilitation and worker health & safety as users can - for the first time - use the device at home or at work, without needing expensive/voluminous specialized equipment (rehabilitation robot or work exoskeleton). The i-Hand glove has a slim design and the same look and feel as a regular glove with a weight under 70g. Mini-actuators and high performance batteries provide enough strength to support activities at work or at home for 8-16 hours without recharge, while keeping the total weight of the system under 400g. The device production cost will be able to reach below 180 Euros at volumes > 500-1000 units. The project will conduct user trials in 3 concrete scenarios (assist, rehab and prevent) in order to gather additional evidence of its benefits and provide end-user iterative feedback. Bioservo has already closed agreements with leading partners such as OttoBock (orthopaedic devices), Hocoma (rehabilitation systems) and has just been contracted by GM, NASA and Airbus for the development of an industrial glove. The glove is already endorsed by key stakeholders such as Swedish Employment Service, the Norwegian Labour and Welfare Administration and the Finnish Health Authority. The project should enable Bioservo to generate 53M Euros and 178 new jobs by 2024.

Evaluation Summary Report

Evaluation Result

Total score: 13.53 (Threshold: 12)

Form information

Indicative Appraisal Scale per Sub-Criterion:
- Very Good to Excellent (4.5 – 5)
- Good to Very Good (3.5 – 4.49)
- Fair to Good (2.5 – 3.49)
- Insufficient to Fair (1.5 – 2.49)
- Insufficient (0-1.49)

Operational Capacity

Status: **Operational Capacity: Yes**

If NO, please indicate the partner(s) concerned, and provide a short explanation. In any case, evaluate the full proposal, taking into account all partners and activities.

Not provided

Criterion 1 - Impact

Score: **4.50** (Threshold: 4/5.00 , Weight: 100.00%)

The expected impacts listed in the work programme under the relevant topic:

The proposal describes in a realistic and relevant way how the innovation has the potential to boost the growth of the applying company.

Good to Very Good (3.5 – 4.49)

Enhancing innovation capacity:

The proposal demonstrates the alignment with the overall strategy of the participating SME(s) and the need for commercial and management experience, including understanding of the financial and organizational requirements for commercial exploitation as well as key third parties needed

Good to Very Good (3.5 – 4.49)

Strengthening the competitiveness and growth of companies and create new market opportunities:

A European added value has been taken into account in the following aspects: a) the assessment of the market, b) the analysis of the competition, c) the impact on EU/global challenges.
Good to Very Good (3.5 – 4.49)

The proposal indicates in a convincing way that there will be demand/market (willingness to pay) for the innovation when the product /solution is introduced into the market.
Good to Very Good (3.5 – 4.49)

The targeted users or user groups of the final product/application, and their needs, are well described and the proposal provides a realistic description of why the identified groups will have an interest in using/buying the product/application, compared to current solutions available.
Very Good to Excellent (4.5 – 5)

Address issues related to climate change or the environment, or bring other important benefits for society (not already covered above):

The proposal adequately addresses issues related to climate change or the environment, or brings other important benefits for society.
Good to Very Good (3.5 – 4.49)

Quality of the proposed measures to exploit and disseminate the project results, and communicate the project activities to different target audiences:

The applicant has made a thorough competition analysis including a) description of competitors and competing products or services, and b) reasons to buy the proposed innovation rather than alternatives.
Very Good to Excellent (4.5 – 5)

The commercialisation strategy is described in a realistic and relevant way, including approximate time to market/deployment. Activities to be further developed after phase 2, including additional dissemination measures, are well outlined.
Very Good to Excellent (4.5 – 5)

Measures to ensure "freedom to operate" (possibility of commercial exploitation) are realistic and there is a convincing strategy of knowledge protection, including current IPR filing status, IPR ownership and licensing issues. Regulatory and/or standard requirements are well addressed.
Very Good to Excellent (4.5 – 5)

Overall assessment of the Impact criterion

(25% weight in the assessment of this criterion)
Very Good to Excellent (4.5 – 5)

Criterion 2 - Excellence

Score: **4.41** (Threshold: 3/5.00 , Weight: 100.00%)

Clarity and pertinence of the objectives:

The objectives for the project as well as the approach and activities to be developed are consistent with the expected impact (commercialisation/deployment). Specifications for the outcome of the project and criteria for success are well defined.
Good to Very Good (3.5 – 4.49)

Credibility of the proposed methodology:

The expected performances of the innovation are convincing and have the potential to be relevant in terms of value for money.
Good to Very Good (3.5 – 4.49)

Soundness of the concept, including appropriate consideration of interdisciplinary approaches and, where relevant, use of stakeholder knowledge:

The proposal reflects a very good understanding of both risks and opportunities related to a successful market introduction of the innovation, from a technical, commercial and regulatory point of view.

Good to Very Good (3.5 – 4.49)

The feasibility assessment (developed under Phase I or through other means) demonstrates the technological/practical/economic viability of the innovation.
Good to Very Good (3.5 – 4.49)

Extent that the proposed work is beyond the state of the art, and demonstrates innovation potential:

With the proposed innovation, the company aims to explore new market opportunities addressing EU/global challenges.
Good to Very Good (3.5 – 4.49)

The current stage of development (TRL 6 - see note 1- or similar for non-technological innovations) is well described. The steps planned to take this innovation to the market are clearly outlined.
Note 1: Please see part G of the General Annexes. (N.B.: In the case of SMEInst-05-2016-2017, the Technology Readiness Levels indication does not apply)
Good to Very Good (3.5 – 4.49)

The proposal makes a realistic comparison with the current state-of-the-art solutions, including costs, environmental benefits, gender dimension- see note 2-, ease-of-use and other features.
Note 2:In relation to the project content, e.g. gender studies, clinical trials, etc.
Very Good to Excellent (4.5 – 5)

Overall assessment of the Excellence criterion

(25% weight in the assessment of this criterion)
Good to Very Good (3.5 – 4.49)

Criterion 3 - Quality and efficiency of implementation

Score: **4.62** (Threshold: 3/5.00 , Weight: 100.00%)

Quality and effectiveness of the work plan, including extent to which the resources assigned to work packages are in line with their objectives and deliverables:

The proposal demonstrates that the project has the relevant resources (personnel, facilities, networks, etc.) to develop its activities in the most suitable conditions. If relevant, describes in a realistic way how key stakeholders / partners / subcontractors could be involved and why and how they were selected (subcontractors must be selected using the best-value-for-money principles). (Where relevant-participants in a consortium are complementary).
Very Good to Excellent (4.5 – 5)

Complementarity of the participants and extend to which the consortium as a whole brings together the necessary expertise:

The team has relevant technical/scientific knowledge/management experience, and a very good understanding of the relevant market aspects for the particular innovation. If relevant, the proposal includes a plan to acquire missing competences, namely through partnerships or subcontracting (subcontractors must be selected using the best-value-for-money principles).
Very Good to Excellent (4.5 – 5)

Appropriateness of the allocation of tasks, ensuring that all participants have a valid role and adequate resources in the project to fulfil that role:

Taking the project's ambition and objectives into account, the proposal includes a realistic time frame and a comprehensive implementation description.
Very Good to Excellent (4.5 – 5)

The work package descriptions and major deliverables and milestones are realistic and relevant, including appropriateness of the allocation of tasks and resources, risk and innovation management.
Very Good to Excellent (4.5 – 5)

Overall assessment of the Quality and Efficiency of Implementation Criterion

(25% weight in the assessment of this criterion)
Very Good to Excellent (4.5 – 5)

Subcontracting

Subcontracting is acceptable in terms of 'best value for money': (only for NON-SMEInst-05-2016-2017: Supporting innovative SMEs in the healthcare biotechnology sector projects) except for task(s):

N.B.: A blank section means either a positive assessment of all your subcontracting tasks or that your proposal does not foresee any subcontracting activities.

Scope of the proposal

Status: **Yes**

Comments:

Not provided

Use of human embryonic stem cells (hESC)

Does this proposal involve the use of hESC?

No

If yes, please state whether the use of hESC is, or is not, in your opinion, necessary to achieve the scientific objectives of the proposal and the reasons why. Alternatively, please also state if it cannot be assessed whether the use of hESC is necessary or not because of a lack of information.

Not provided

Overall comments

Not provided

Digitally sealed by the European Commission
Date: 2017.05.16 17:37:08 CEST

This document is digitally sealed. The digital sealing mechanism uniquely binds the document to the modules of the Participant Portal of the European Commission, to the transaction for which it was generated and ensures its integrity and authenticity.

Any attempt to modify the content will lead to a breach of the electronic seal, which can be verified at any time by clicking on the digital seal validation symbol.
